# Application of Crack Identification Techniques for an Aging Concrete Bridge Inspection Using an Unmanned Aerial Vehicle

**DOI:** 10.3390/s18061881

**Published:** 2018-06-08

**Authors:** In-Ho Kim, Haemin Jeon, Seung-Chan Baek, Won-Hwa Hong, Hyung-Jo Jung

**Affiliations:** 1 Applied Science Research Institute, Korea Advanced Institute of Science and Technology, Daejeon 34141, Korea; kih119@kaist.ac.kr; 2 Department of Civil and Environmental Engineering, Hanbat National University, Daejeon 34158, Korea; 3 Division of Electronics and Info-Communication Engineering, YeungJin College, Daegu 41527, Korea; baeksc@yjc.ac.kr; 4 School of Architecture, Civil, Environmental and Energy Engineering, Kyungpook National University, Daegu 41566, Korea; hongwh@knu.ac.kr; 5 Department of Civil and Environmental Engineering, Korea Advanced Institute of Science and Technology, Daejeon 34141, Korea

**Keywords:** crack identification, deep learning, unmanned aerial vehicle (UAV), computer vision, spatial information

## Abstract

Bridge inspection using unmanned aerial vehicles (UAV) with high performance vision sensors has received considerable attention due to its safety and reliability. As bridges become obsolete, the number of bridges that need to be inspected increases, and they require much maintenance cost. Therefore, a bridge inspection method based on UAV with vision sensors is proposed as one of the promising strategies to maintain bridges. In this paper, a crack identification method by using a commercial UAV with a high resolution vision sensor is investigated in an aging concrete bridge. First, a point cloud-based background model is generated in the preliminary flight. Then, cracks on the structural surface are detected with the deep learning algorithm, and their thickness and length are calculated. In the deep learning method, region with convolutional neural networks (R-CNN)-based transfer learning is applied. As a result, a new network for the 384 collected crack images of 256 × 256 pixel resolution is generated from the pre-trained network. A field test is conducted to verify the proposed approach, and the experimental results proved that the UAV-based bridge inspection is effective at identifying and quantifying the cracks on the structures.

## 1. Introduction

Continuous safety monitoring and maintenance of infra-structure such as bridges are essential issues. The effects such as fatigue load, thermal expansion, contraction and external load decrease the service performances of bridges overtime. As bridges become obsolete, the number of bridges that need inspection increases, and this requires much maintenance cost. If we postpone spending on bridge maintenance, more costs will be required in the near future. Thus, many countries established a maintenance plan of bridges. As a crack directly reflects the condition of structures, it is considered as one of the important parameters for structural health monitoring. Conventional crack detections are carried out by human visual inspection. This method has limitations that the performance is highly related to the experience of the inspector, time consumption and limited accessible areas [1].

Some approaches including the use of a charge-coupled device (CCD) camera [2,3], a complementary metal oxide semiconductor (CMOS) image sensor [4,5], near-infrared (NIR) [6,7] and a hyper-spectral camera [8,9] have been studied. In most of the studies, the image processing consists of the following steps [10,11,12]: (1) image acquisition; (2) pre-processing techniques, which are methodologies for efficient image processing; (3) image processing techniques such as binarization, noise elimination using a mask filter or morphological processing; and (4) crack quantification, which is a parameter estimation of the crack. Image binarization methods are implemented for converting an RGB image to a binary image. Talab et al. [13] proposed a simple algorithm that was compared to other binarization methods to detect cracks. Kim et al. [14] represented issues in image binarization for crack identification and proposed the hybrid image processing method. The proposed method is composed of image pre-processing such as image undistortion and crack thickness estimation. Edge detection techniques, which are mathematical methods to find rapid changes of image brightness, were proposed [15]. Abdel-Qader et al. [16] considered and compared four edge-detection algorithms, which were fast Haar transform, fast Fourier transform, Sobel and Canny. Image processing-based crack detection algorithms show effective performance, and an alternative method to human visual inspection can be shown. However, many issues for vision-based bridge inspection must necessarily be worked out to implement automated crack detection. In practice, low contrast between cracks and the surface of the structure, intensity inhomogeneity and shadows with similar intensity to the crack make automatic crack detection difficult. Zou et al. [17] proposed CrackTree, an automatic crack detection method from pavement images. The procedure of the proposed method is composed of shadow removal, constructing a crack probability map, crack seeds and crack detection. They focused on detecting the location and shape of the crack without quantification. Li et al. [18] proposed FoSA, which is the F* seed-growing approach for crack line detection. Shi et al. [19] investigated CrackForest, which is random structured forest-based automatic road crack detection. As described above, various algorithms have been proposed and validated for crack detection. However, other methods are required because the cracks in bridges can also occur in inaccessible members.

In recent years, bridge inspection based on unmanned aerial vehicles (UAV) with vision sensors has received considerable attention in many countries due to its safety and reliability. A UAV equipped with a camera was used to store digital images taken during crack detection through scanning the surface of the bridge. Since it is efficient, fast, safe and cost-effective, local departments of transportation (DOT) in the United State have been investigating and starting to apply the UAV-based bridge inspection technique [20]. In a report by the American Association of State Highway and Transportation Officials (AASHTO), 16 state DOTs use UAV for bridge inspection. However, they roughly evaluate damage conditions without knowing its size and location [21]. A GPS-denied environment under a bridge decreases the stability of the UAV platform. Thus, many flight planning methods using appearance image-based recognition [22], simultaneous localization and mapping (SLAM) [23], Lidar odometry mapping in real-time (LOAM) [24], etc. [25], have been proposed to reduce GPS error. Other researchers proposed and investigated a protocol for bridge inspection to address the challenges [14,26,27].

Recently, deep learning-based crack detection methods have been proposed [28,29,30]. The features are extracted from training the crack images by a convolutional neural network. The results of crack detection using deep learning overcome the limitation of conventional image processing techniques such as blob and edge detection. To localize and visualize the detected crack, a framework for an integrated building information model (BIM) with a UAV process was investigated. Its advantages are that it is easy to visualize the structural elements and to manage maintenance history. However, there are many bridges that do not have a BIM, and deriving a new BIM takes much time and effort. As such, the UAV-based crack detection system is in the embryonic stages, requiring solutions for the many challenging issues in its practical applications. The accuracy of the localization of a UAV in GPS shaded areas such as bridge decks is one of the primary concerns [31]. Moreover, longer flight time and more stable flight are required for stable inspection. In approaching the bridge, it is essential to ensure that the field of view (FOV) determines the pixel size of the image. Keeping both the FOV and pixel size is critical because the quantification standard depends on pixel size.

In this paper, a crack identification method via image processing taken by a commercial UAV with a high resolution camera is investigated for an aging concrete bridge. Specifically, the framework and challenge are provided to identify the appearance of cracks on the bridge using UAV. The inspector can create a background model on a bridge with no information. He/she also can track the damage history by visualizing cracks on the inspection map. The detailed process of this study is as follows. First, the point cloud-based background model of the bridge was generated in a preliminary flight. Then, inspection images from a high resolution camera mounted on UAV were captured and stored to scan structural elements. Finally, we conducted prerequisite, deep learning processing for both image classification and localization; and crack size estimation to quantify the cracks. To quantify the cracks on captured images, reference markers were attached to the structures in advance. In the deep learning method, the regions with convolutional neural networks (R-CNN) method that significantly reduces the computational cost has been used for crack detection and finding its location. Finally, the length and thickness of the crack is calculated using various image processing techniques and validated through the field test.

## 2. Proposed Methodology

### 2.1. Overview

**Figure 1:**
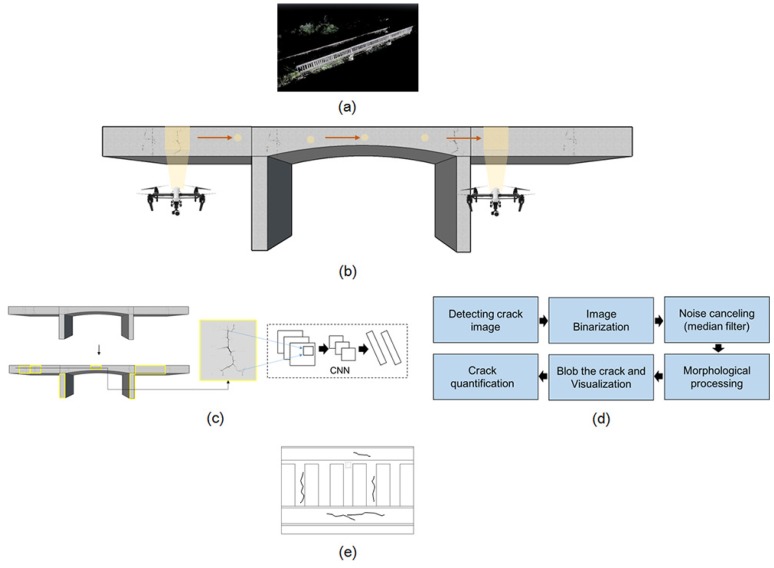
Overview of the UAV-based crack identification: (**a**) Generating background model; (**b**) Image acquisition to scan appearance status; (**c**) Crack detection using deep learning algorithm; (**d**) Crack quantification using image processing and (**e**) Visualization of identified crack on the inspection map.

Figure 1 shows a schematic of the crack identification using UAV. As shown in this figure, UAV-based crack identification consists of five main phases: (1) the generation of the point cloud-based background model to build a damage map by visual inspection (inspection map); (2) image acquisition using a camera mounted on a UAV; (3) crack detection using the deep learning method; (4) crack quantification using image processing; and (5) visualization of the identified crack on the inspection map. The proposed approach collects and stores imaging data by using camera-equipped UAV from the region of interest (ROI) on the bridge. The preliminary study to visualize crack information on the inspection map was conducted.

### 2.2. Background Model Generation for Building the Spatial Information

**Figure 2:**
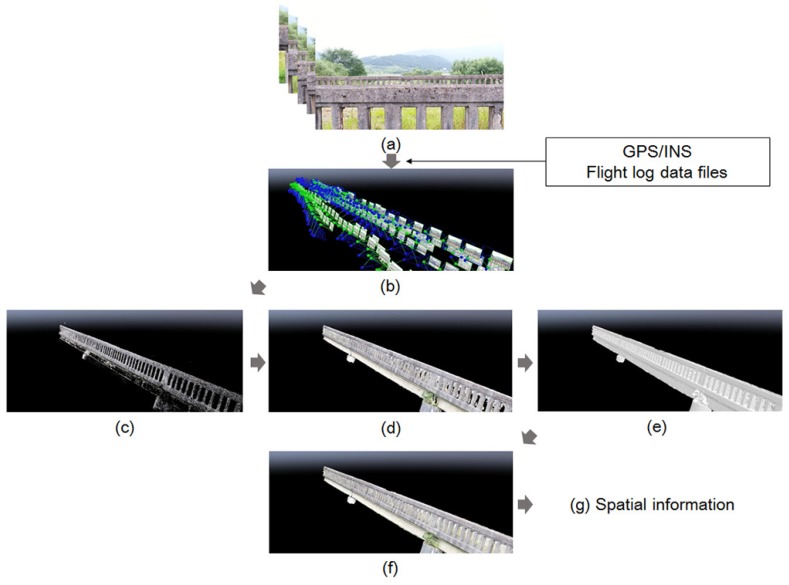
Generating background model to build spatial information: (**a**) Aerial images; (**b**) Geotagged imaged; (**c**) Tie points matching; (**d**) Point clouding; (**e**) Mesh building; (**f**) Texturing and (**g**) Spatial information.

The first phase of crack identification is the generation of the background model using UAV. Detecting and quantifying the cracks and representing the information on the map is essential for recording and utilizing the inspection results [32]. Since most aging bridges have no information about the shape of the structures [33], we generated the inspection map for selected bridges to store the crack information in a database. To build the map using aerial photographs, the flight altitude, interval photographing and overlap ratio should be considered. As the flight altitude is related to the spatial resolution of the image, the height of the UAV should consider an effective resolution for the camera performance. The overlap ratio related to the shutter speed is recommended to be more than 60% for the longitudinal and 30% for the lateral direction. 3D vectorization of images is required to consider the scale of the inspection map because the aerial images obtained by UAV are 2D raster images. In this study, the 2D model based on the stitching method could be utilized as the same side of the bridge was inspected. However, the 2D image matching method has the drawback that it is difficult to create a digital orthoimage, as well as the occlusion region. Therefore, the point cloud technique was applied to build a 3D point cloud model and 3D spatial information by referring to the point information extracted from the 2D images [34]. The procedures of making an inspection map based on the point cloud are shown in Figure 2 [35,36]. The UAV stored GPS and inertial navigation system (INS) information. This information (also called metadata) was combined with the images using the geo-tagging process at first. Then, the coincident points were extracted from the overlapping region of geo-tagged images. The number of extracted coincident points was set to adjust the tolerance. The point cloud data having the position information were formed as a result of the cross-reference to the overlap region for the extracted coincidence. Finally, 3D modeling was conducted through the mesh formation process based on the point cloud data. As a result, the 2D bridge spatial information was generated by mapping the texture as the original image to the generated 3D model [37]. Commercial software, Pix4D Mapper, has been used for 3D model generation in this paper. The spatial information was converted into digital design information by using AutoCAD 2017.

### 2.3. Image Acquisition with a High Resolution Camera on the UAV

**Figure 3:**
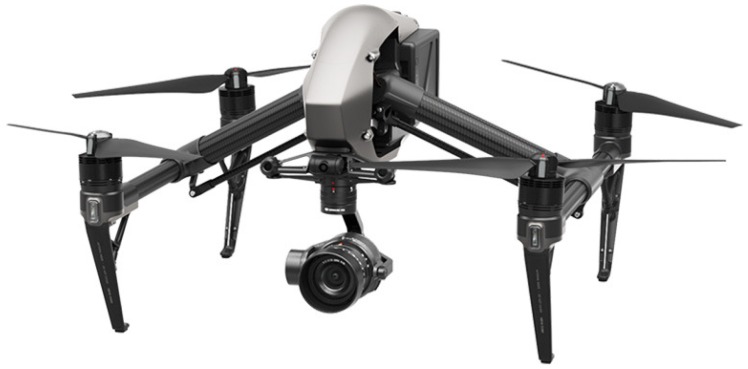
Inspire 2 with Zenmuse X5S for crack identification.

The flight of the UAV at the bridge should consider the safety as the priority. Many guidelines were suggested to ensure safety, but the core contents will be presented in this section. At first, the UAV must fly within a flight altitude of 150 m to avoid collisions with aircraft. The UAV should also refrain from flying over the vehicle in the case of a fall. Finally, the operation speed of the UAV should be limited to 80% of the maximum speed to cope with an unexpected situation such as denied GPS and gusts of wind around the bridge. It is very important to keep the distance between the UAV and the target element to collect images because the pixel size for quantification can be calculated by the exact focal distance. However, it is difficult to keep the focal distance in flight, so the localization of UAV is one of the major concerns in bridge inspection due to denied GPS around the bridge. Therefore, planar markers were used as references to calculate the pixel size of images without knowing the location of the UAV and the distance from the target in this study. Several markers with known sizes were attached on the surface of the bridge. The UAV (i.e., Inspire 2) and a vision sensor (i.e., Zenmuse X5S) used in this study were manufactured by DJI Co., Ltd. (Shenzhen, China), and the two systems were connected by a gimbal, as shown in Figure 3. The gimbal could tilt the camera up from $-130^{{}^{\circ}}$ to +$40^{{}^{\circ}}$, pan $320^{{}^{\circ}}$ and roll $20^{{}^{\circ}}$ in either direction. The UAV type was a rotary-wing, and the resolution of the camera was 5280 × 2970 pixels when the aspect ratio was 16:9. The flight control was manually operated, and the UAV flew while scanning the appearance of the bridge and keeping its distance at approximately 2 m from the concrete deck.

### 2.4. Crack Detection Using Deep Learning

This section describes the crack detection using deep learning method. In this paper, crack detection was defined as performing both classification and localization for an image. Many researchers have proposed automated crack detection using image processing and pattern recognition techniques. In most studies, crack features in the captured image were extracted and matched using the image processing techniques, such as blob detection, edge detection and the support vector machine (SVM) algorithm. Meanwhile, a sliding window technique with an image pyramid algorithm has been proposed. A sliding window scans an image sequentially, and an image pyramid algorithm reduces the size of the input image step by step. Therefore, the objects are effectively detected for various scales and locations.

**Figure 4:**
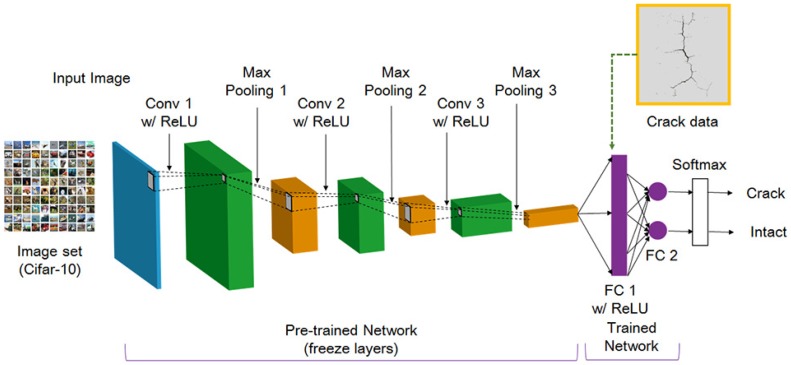
Schematic of the deep learning architecture.

Recently, a sliding window-based CNN, one of the deep learning methods, was proposed, and its performance was validated for object classification [38,39,40]. However, the CNN has disadvantages such as a high computational cost and a long operation time. To overcome these drawback, the region proposal algorithm was proposed to quickly scan the meaningful region in the image. In this paper, the R-CNN method combined region proposals with rich features extracted by CNN and was applied to detect the cracks. The steps of R-CNN consisted of extracting the region proposal from the input image using selective search, feature extraction by using CNN after cropping and image detection after bounding-box regression [41]. In the feature extraction step, insufficient data made it difficult to train the features. Therefore, the transfer learning method is commonly used in deep learning applications. The concept of transfer learning is to use features and parameters extracted from a large image set with many labeled data when the labeled data are insufficient. This method conducts the training of a large image set such as ImageNet [42] and Cifar-10 [43] in advance. Then, new dataset, which was comprised crack data in this study, is connected with the pre-trained network by using fine-tuning. Figure 4 presents the deep learning architecture to extract the feature for crack detection in this study. The input layer is 50,000 image of 32 × 32 × 3 pixel resolution in the Cifar-10 image set, which is large image dataset of 10 classes. In the convolution layer, 32 filters (also called the kernel) with a size of 5 × 5 were used, and a stride of 1 and a padding of 2 to prevent image downsizing. The ReLU as a nonlinear activation function was used because of its fast computation and high accuracy [44]. The max pooling with 3 × 3 kernel and a stride of 2 was used. The fine-tuning was applied to the fully-connected layer to classify the crack. Table 1 lists the detailed information of each layer.

### 2.5. Image Processing for Crack Quantification

**Figure 5:**
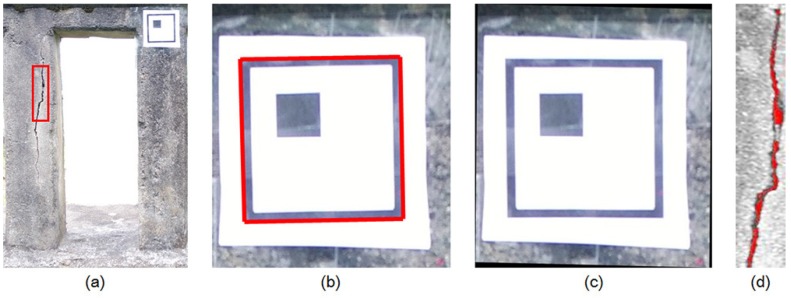
Image processing procedure of crack length and thickness estimation: (**a**) Image capture; (**b**) Marker detection; (**c**) Calculation for fixel size and (**d**) Crack quantification.

To quantify detected cracks, the total length and thickness have been calculated using a planar marker. By using the relationship between the given 3D points in the world frame and their corresponding points in the captured image, the homography transformation matrix could be calculated. From the calculated matrix, the distorted image could be corrected. The procedure of estimating the crack’s length and thickness using a planar marker is shown in Figure 5. The camera captured the image including the marker, as shown in Figure 5a, then the marker was detected by using the region of interest (ROI) algorithm with the Sobel edge detection algorithm (see Figure 5b). After finding four corners of the marker, the transformation matrix could be estimated and was applied to the distorted image. From the undistorted image, as shown in Figure 5c, the diagonal length and its thickness can be calculated, as shown in Figure 5d. To detect the horizontality and verticality of edges that can be defined as cracks, the detected image was rotated in the longitudinal direction, and image convolution was performed with the following mask: **h** = [−1 −1 −1; 1 1 1]’.

## 3. Experimental Validation

**Figure 6:**
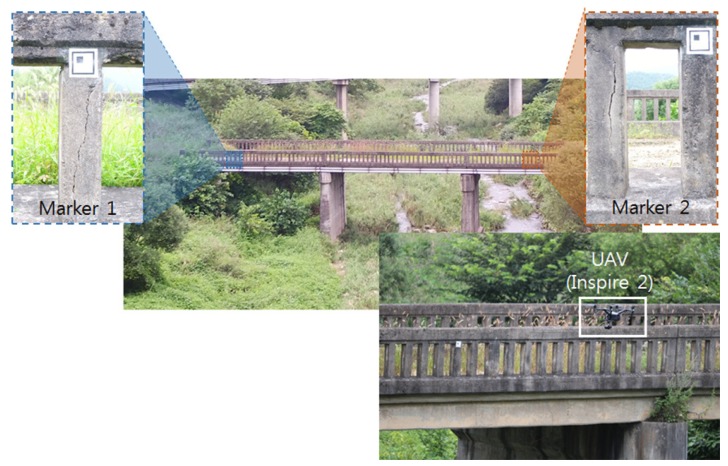
Experimental setup.

A field test was conducted to verify the proposed crack detection method based on the UAV with an imaging device in an old concrete bridge. In this study, target elements in bridge were examined for UAV-based visual inspection. At first, the inaccessible elements such as abutments, piers and pylons could be targeted. The bridge deck could be included if the camera were pointing up through the gimbal. The bridge rails were only targeted in this study, but further study will cover other elements. In the experiment, the UAV made a flight for two purposes (i.e., generating the background model and image acquisition for crack detection), and different flight methods were applied. The flight for generating the background model considered driving repetition number and image overlap rate, and the camera mounted on the UAV took a picture along the path. On the other hand, the flight for inspection considered the scanning speed, FOV and dynamics, focusing on obtaining high-quality images. Figure 6 shows the experimental setup for the crack detection using the UAV. Two markers were attached on the bridge to quantify crack size because it is challenging to exactly know both the location of the UAV and the distance from the target. Further study will be conducted to address the challenges without the marker. In this experiment, a square-shaped planar marker was designed and applied. Thus, the pixel size and rotational angle could be estimated from the marker.

### 3.1. Generating the Background Model

In this section, the procedure of the background model generation using captured images from the UAV is described. In this paper, a closed bridge with much crack damage was selected.

A total of 277 images were obtained from a high resolution camera mounted on the UAV during the flight time of 11 min. When constructing the spatial information using the point cloud technique, it is required to secure vertical aerial photographs between each image. The vertical aerial photographs were a set of over 70% in this study. Flight control was manually operated to consider the path plan. As a result of checking the obtained images, the outline of each structure member in the bridge was clearly revealed. Furthermore, the UAV can hardly achieve the same vertical aerial photographs between the images due to the manual flight, but can achieve over 70% vertical aerial photographs.

**Figure 7:**
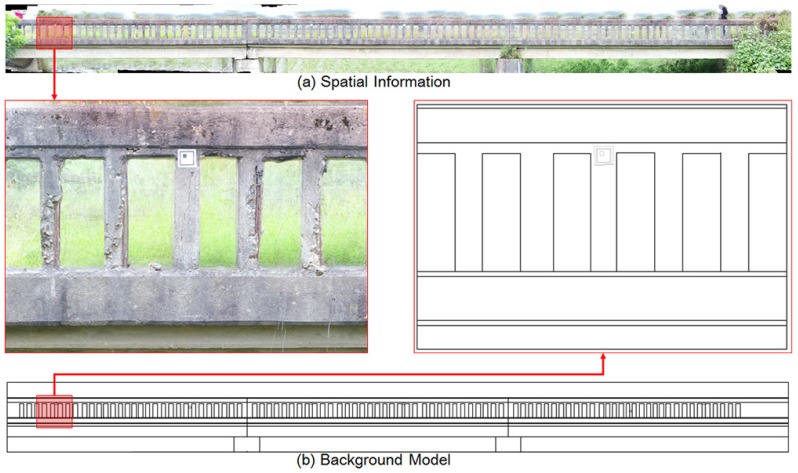
Final results for spatial information and the background model.

The vision sensor mounted on UAV captured the image of the structures, then the 3D point cloud model of the bridge was generated with the use of GPS data. Finally, the detected cracks were visualized on the digitized inspection map. In this study, 41,974 tie points between each image were extracted, and the orthoimagery of ground sampling distance (GSD) 0.10 cm/pixel level was generated as shown in Figure 7. Figure 7a represents the spatial information of the bridge, and Figure 7b shows the general drawing of the bridge in order to utilize the inspection map.

### 3.2. Crack Detection

**Figure 8:**
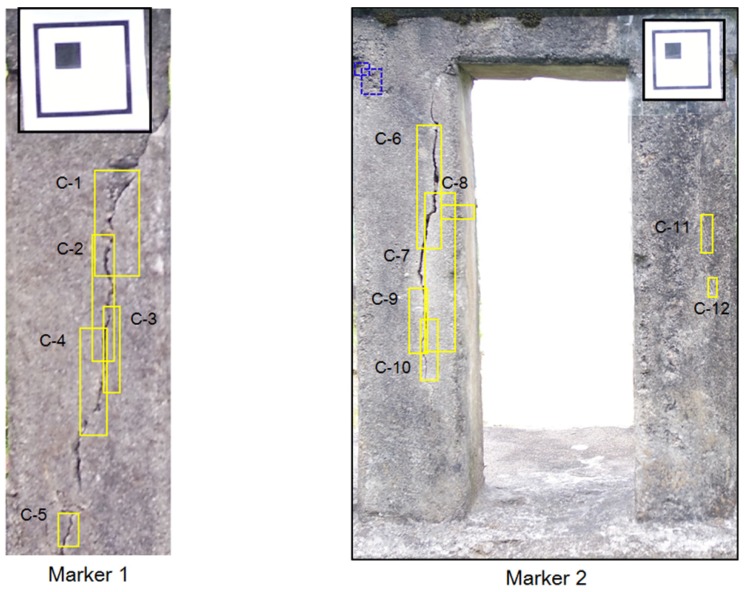
Results of crack (C) detection.

In this paper, the Cifar-10 dataset, which has 50,000 images of 32 × 32 RGB pixels for a pre-trained network, and 384 crack images of 256 × 256 RGB pixels for fine-tuning have been used. To collect the training data for the deep learning, both methods using man-power and the UAV were utilized in concrete bridges and structures without the target bridge. Some images were collected on the web. In order to reduce the imbalance in the crack direction and increase the image, a method of rotating a part of the collected images was used. Then, a labeling process was manually performed on the cracks in the image. The CNN training algorithm using Cifar-10 data used the stochastic gradient descent with momentum (SGDM). The initial learning rate was set to be 0.001, and it was reduced every 8 epochs during 40 epochs. Ten classes of Cifar-10 were changed to the crack class with the background through transfer learning. Then, the bounding box regressions to improve the accuracy of region proposals were conducted after training one binary SVM per class to classify the region features. In this study, two regions of interest including both the crack and marker were specified and cropped. Figure 8 shows the results of crack detection by using deep learning. In the figure, there are water marked rectangle boxes, which show the accuracy of the results. The labeled boxes are detected cracks (C) obtained by the deep learning process. The dotted boxes are also detected cracks, but they do not seem like cracks due to blurring. These results show that the deep learning method has better detection performance than the traditional image processing techniques. However, the parameters of the selected region should be modified so that the box can contain the crack size. In this study, the deep learning was trained considering the direction of cracks. In addition, training data were acquired through image rotation to reduce the unbalance between vertical crack and horizontal crack data. As a result, the deep learning model was able to detect the horizontal crack, C-8. Therefore, the proposed approach can be validated to conduct the tasks well, which are classification and localization of cracks.

### 3.3. Crack Quantification

**Figure 9:**
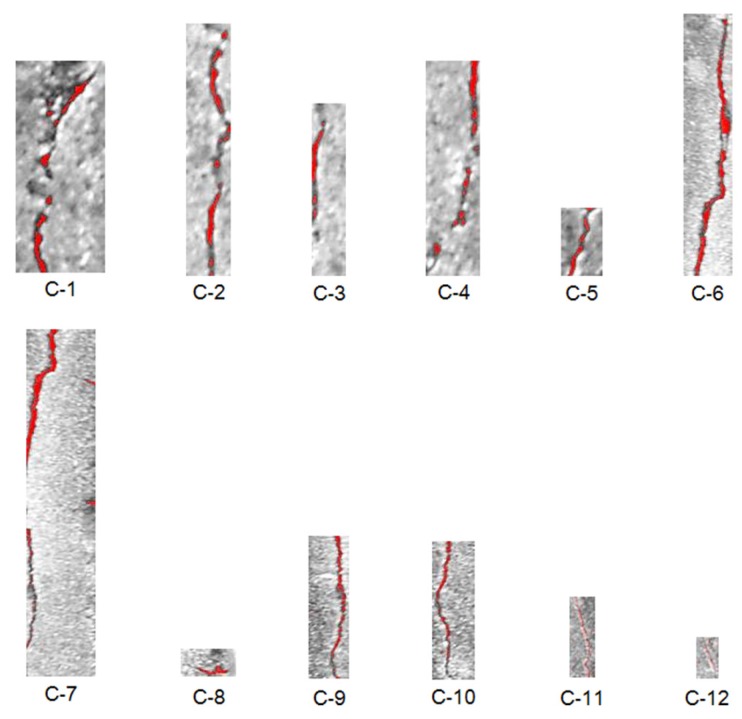
Crack quantification by using image processing.

In this section, the quantification of the detected and cropped cracks is described. To quantify the cracks, it is necessary to know the exact pixel size in the image. UAV-based systems can use GPS data and focal distance to predict pixel size, but there is an error that affects the resolution. To estimate pixel size, planar markers sized 70 × 70 mm were attached on the structure, and the vision sensor mounted on the UAV captured the structure including the marker and cracks. In the field test, the conventional square-type marker has been used. The procedure of estimating the length and thickness estimation were identical to as described in Section 2.3. The crack quantification algorithm, which was verified in a small-scale laboratory test that provided a relative error of 1∼2%, has been used to calculate length and thickness. Figure 9 shows the results of the marked crack by using the image processing method, and Table 2 shows the estimated results. In this study, the crack quantification based on image processing was considered for the bounding boxes. From the results, it was possible to quantify cracks, but it was difficult to estimate information on some cracks (i.e., C-11 and C-12). Because the dark surface of the surrounding crack makes quantification of the crack hard work, two cracks were hardly quantified. The crack quantification algorithm based on image processing could be affected by the surrounding environment such as shadows and the intensity and direction of sunlight. Some of these problems could be addressed by considering the lighting equipment and post-processing method.

### 3.4. Bridge Inspection Results

**Figure 10:**
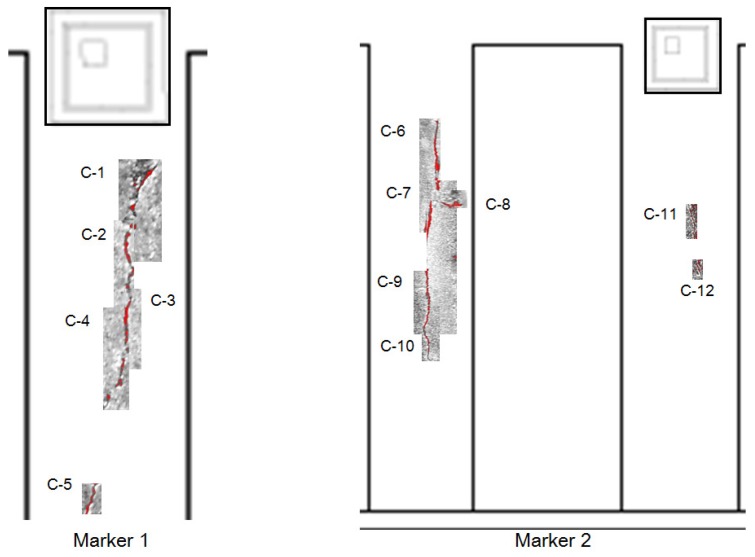
Bridge inspection results.

In the previous section, we confirmed that the thickness and length of cracks can be quantified via image processing. The coordinates and area of the detected crack, the quantified crack information, can be manually displayed on the inspection map as shown in Figure 10. The cracks expressed in the inspection map were realistic and could be stored and compared with later inspection results. We are investigating ways to link the results to the bridge management system (BMS) and to be processed automatically in the future.

## 4. Discussion

The UAV-based crack detection method has received considerable attention because of its stability and reliability. However, this technology is still considered to be in the primitive stages because there are many challenges to be solved for practical applications. We looked over the whole UAV-based crack identification process from generating the background model to crack visualization on the inspection map. The UAV flight and crack inspection were conducted in a real civil structure. The detected crack size was calculated by using the installed 2D marker because the accuracy for localization of the UAV should be improved. In the crack detection, R-CNN was used for deep learning, and 384 crack images were combined with the pre-trained model through transfer learning. As a result, satisfactory performances for both classification and localization were validated. The optimization for the deep learning model and a rich dataset for cracks could effectively improve the performance. In this study, the coordinate values and locations of bounding boxes for detected cracks were obtained, and the detected boxes were automatically cropped and stored. Each cropped image could be quantified through image processing and visualized on an inspection map at the same scale. In the process of quantification of the detected cracks, there was difficulty in the quantification of some cracks. Aging concrete bridges may have contamination on the surface. In addition, the image processing techniques for crack quantification can be affected by the surrounding environment, such as shadows and the intensity of sunlight. Therefore, it is necessary to solve these problems through lighting equipment and post-processing.

To identify and visualize cracks, a high performance computer composed of a CPU (i.e., i7-7700, Intel, Santa Clara, CA, USA), RAM memory of 32.0 GB and a GPU (i.e., Geforce GTX 1080 Ti, NVIDIA, Santa Clara, CA, USA) has been used. Regarding the computation time of generating the background model of the bridge, the 3D point cloud process using the Pix4D mapper was approximately 150 min, and the bridge information extraction using AutoCAD required another 30 min. In the deep learning, approximately 14 min were required to conduct crack detection including the network training. Finally, the computation time for quantification and visualization of cracks was about 5 s. In a large bridge, the computation time will increase as the bulk of images are processed.

## 5. Conclusions

In this paper, the applications of the UAV with the deep learning algorithm in crack identification were presented. The study focuses on processing with collected images to identify the cracks of a concrete bridge. The crack identification and visualization method was applied to a real civil bridge. Because most of the aging bridges have no inspection map, the 3D point cloud-based background model was considered. The background model generated in the preliminary flight has been used for the visualization of the identified crack on the inspection map. In this study, preliminary and inspection flight mode were considered, and the guidelines for flight were presented. To detect the crack, the deep learning algorithm was used to effectively conduct the tasks of both image classification and localization. The R-CNN instead of CNN-based on a sliding window was used as the object detector, and transfer learning with a rich dataset was used in the deep learning architecture. Fifty thousand training images from the Cifar-10 dataset were pre-trained using CNN. Then, the pre-trained network was fine-tuned for crack detection using the 384 collected crack images. In object detection, various cracks could be detected by training the deep learning network with a small number of crack images. The detected cracks were cropped and quantified by image processing. Finally, the identified cracks were automatically visualized on the inspection map using matching of their location. In this study, a field test to apply crack identification techniques in the aging bridge was conducted. As a result, the performance of the proposed technique has been validated to effectively inspect cracks, and the UAV-based bridge inspection system could be considered as one of the promising strategies.

## Figures and Tables

**Table 1:** Information of each layer of the deep learning architecture.

Layer	Operator	Dimension (Height × Width × Depth)	Kernel (Height × Width)	Stride	Padding
Input	Conv 1 w/ReLU	32 × 32 × 3	5 × 5	1	2
Layer 1	Pool 1	32 × 32 × 32	3 × 3	2	0
Layer 2	Conv 2 w/ReLU	16 × 16 × 32	5 × 5	1	2
Layer 3	Pool 2	16 × 16 × 32	3 × 3	2	0
Layer 4	Conv 3 w/ReLU	8 × 8 × 32	5 × 5	1	2
Layer 5	Pool 3	8 × 8 × 64	3 × 3	2	0
Layer 6	-	4 × 4 × 64	-	-	-
Layer 7	FC 1	1 × 1 × 64	-	-	-
Layer 8	FC 2	1 × 1 × 2	-	-	-
Layer 9	Softmax	1 × 1 × 2	-	-	-

**Table 2:** Results of crack quantification.

	Crack Thickness (mm)	Crack Length (mm)
C-1	1.92	48.68
C-2	1.10	60.09
C-3	1.10	27.94
C-4	1.37	48.59
C-5	1.37	17.08
C-6	1.92	63.56
C-7	2.47	78.43
C-8	1.59	6.60
C-9	1.10	35.01
C-10	0.53	30.79
C-11	0.55	19.96
C-12	0.55	8.32
